# An Investigation on the Academic Burden of Chinese Students Ranging from Primary Schools to Universities Based on a Word Association Test in Guangdong Province

**DOI:** 10.3390/ijerph19042481

**Published:** 2022-02-21

**Authors:** Ruixiang Gao, Tingxin He, Yu Liao, Xiaoqin Liu, Yinqing Fan, Yingting Su, Huang Zuo, Lei Mo

**Affiliations:** 1 Philosophy and Social Science Laboratory of Reading and Development in Children and Adolescents (South China Normal University), Ministry of Education, Guangzhou 510631, China; ruixianggao@m.scnu.edu.cn (R.G.); 2020023472@m.scnu.edu.cn (T.H.); lyscnu2022@163.com (Y.L.); yinqing_fan@163.com (Y.F.); 2 School of Psychology, South China Normal University, Guangzhou 510631, China; 3 School of Foreign Studies, South China Normal University, Guangzhou 510631, China; 20182322005@m.scnu.cn; 4 Shanghai Mental Health Center, Shanghai Jiao Tong University School of Medicine, Shanghai 200030, China; 5 School of Mathematics (Zhuhai), Sun Yat-sen University, Zhuhai 519080, China; suyt25@mail2.sysu.edu.cn; 6 Teacher Education and Teacher Development Evaluation Research Center of Guangdong-Hong Kong-Macao Greater Bay Area, College of Teacher Education, South China Normal University, Guangzhou 510631, China

**Keywords:** academic burden, students from primary schools to universities, measurement method, word association test

## Abstract

China’s basic education and higher education are currently facing policies aimed at reducing and increasing the academic burden, respectively. In this context, we first review and assess the methods of measuring students’ academic burden and then apply the implicit association test for the first time to the academic burden of Chinese students from primary school to university under a unified framework. The results demonstrates that students’ academic burden increases with the school stage, and thus university students face a greater burden than primary and high school students, and that learning attitude fully mediates the relationship between objective and subjective views of academic burden. These results suggest the three policy approaches of implementing a management system for classifying academic burden, considering how to improve students’ learning quality, and developing their mental health education, thus providing a reference and inspiration for research and practice in the field of academic burden.

## 1. Introduction

Academic burden is an issue of particular significance in China [1], and it has long been a concern of educational professionals and society [2]. Regarding basic education, the heavy academic burden of primary and high school students has been discussed since the founding of the People’s Republic of China. Various measures to “reduce the burden” [3,4] have been introduced nationally, and extensive research into reducing students’ academic burden has been conducted. CNKI includes 3631 educational research papers on the topic of “reducing the burden” from between January 2010 and December 2020, which amounts to more than 200 publications per year [5]. In May 2021, the 19th Central Commission for Comprehensively Deepening Reform approved what are regarded as the “strictest measures in decades”: the *Opinions on Further Reducing the Burden of Homework and Off-Campus Training for Compulsory Education Students*, which demonstrate the country’s unprecedented determination to reduce the burden in basic education [6]. However, a different approach has been taken with higher education. At the Ministry of Education’s National Conference on Undergraduate Education in Higher Education Institutions in the New Era, Chen Baosheng, the then-Minister of Education, proposed that while reducing the burden on primary and high school students, it was reasonable to “increase the burden” on university students [7]. Further academic challenges, such as increasing the difficulty and depth of courses could thus reverse the phenomenon of “hard-working in high schools, relaxed in universities” in China. In the same year, the Ministry of Education issued the *Notice on* *the Implementation of the Spirit of National Conference on Undergraduate Education in Higher Education Institutions in the New Era*, which identifies 11 “strict” characteristics that can be applied to improve the quality of undergraduate education [8]. Thus, the reduction of the academic burden in basic education and the corresponding increase in higher education have again made it the broader focus of attention in society.

In this context, we address the research question of whether it is appropriate to propose to increase the burden on university students while advocating a reduction in the burden on primary and high school students. Is there any empirical evidence for “hard-working in high schools, relaxed in universities”? We address this question by conducting a survey of university students’ perceptions of academic burden and comparing the burdens of basic and higher education students under a unified framework. Few other studies have been conducted in this area, mainly because the method of measuring academic burden in basic education cannot be directly transferred to higher education. To make a useful comparison under a unified framework, we must first identify a method of investigating university students’ academic burden.

Therefore, we first review the measurement methods identified in the research and then analyze the current situation. We assess the academic burden of Chinese students using empirical data collected for our previous research [9,10], which were obtained from a large-scale survey of learning concepts conducted with students from primary schools to universities in Guangdong Province based on the Word Association Test (WAT). We then explore effective methods of reducing and increasing this burden and offer suggestions that can inform both research and practical policies in the field of academic burden.

## 2. Reviews on Measurement Methods of Students’ Academic Burden

### 2.1. The Definition of Academic Burden

The particular understanding of academic burden will determine how it is measured [11], and quite different views of the concept have been put forward [12]. However, through extensive and in-depth discussions, consensus has gradually been reached.

In terms of the extension of the concept, many similar terms, such as “academic burden”, “learning burden”, and “student burden”, have been applied in government documents, public exchanges, and academic discussions. Obvious contradictions between these concepts have been identified and the relationships between them disputed. Some scholars have lumped them together, while others have distinguished them [13]. The term “academic burden” has recently been accepted as a broad concept, thus preventing endless analyses of the minor differences between similar concepts.

The concept has been the subject of two main controversies regarding its connotations. First, is academic burden a neutral description without any value judgment [14], or a negative expression that implies “exceeding” or “excessive” [15]? This is less of an issue as researchers have begun to use operational definitions [16]. The second question is whether the academic burden can be quantified as an objective number of learning tasks, or does it involve students’ subjective psychological experience? Clearly, it is not appropriate to only understand the academic burden as an external and objective encumbrance for students. Under the same level of tasks, different students have different perceptions about the burden that they shoulder. Thus, both objective and subjective attributes should be considered, and students’ subjective feelings about their burden are likely to merit more attention. Any measures to reduce this burden should therefore not only focus on objective factors but also consider other core variables that may lead to subjective perceptions of pressure. Only then can any initiatives to reduce this burden be effective [17,18,19,20]. In terms of the research approach to academic burden in China, objective and subjective factors typically have been investigated together [21,22,23,24,25]. In this study, we also address both objective and subjective aspects of academic burden.

### 2.2. Progress and Limitations of Research on Objective Academic Burden

Research into academic burden typically applies self-designed questionnaires. Questions concerning the objective factors are typical in the most authoritative works, which involve domestic and international large-scale education assessment projects as references (for example, the Third-Party Evaluation of Projects on Compulsory Education Development and the Compulsory Education Quality Monitoring Program in China, and international projects include TIMSS and PISA, etc.). These mainly involve indicators, such as school time, weekly class hours, exam frequency, homework time, extracurricular tutoring investment, and sleeping time [26,27,28,29]. However, such questions cannot be used to assess the academic burden of university students because the study approaches in higher education and basic education are very different and no specific national policy serves as a “baseline” standard for comparison.

However, some research in the area of higher education has been conducted. Since the early 2000s, notions of national strength have become centered on competition in terms of academic excellence [30,31]. Quality assurance mechanisms for the learning and development of students in higher education have thus been established and developed in various countries [32,33]. In this context, extensive academic research into student engagement has been conducted, and numerous large research projects have emerged in China and elsewhere. For example, the National Survey of Student Engagement (NSSE) and the Student Experience at the Research University (SERU) in the U.S., the Australasian Survey of Student Engagement (AUSSE) in Australia, and the China College Student Survey (CCSS) initiated by Tsinghua University and the National College Student Survey (NCSS) initiated by Xiamen University in China have comprehensively examined university students’ learning and development and the influencing factors [34,35]. These studies all involve identifying objective factors for the academic burden faced by students in higher education to varying extents, but no study has directly assessed such factors.

China’s basic education and higher education are currently facing the policies of reducing and increasing the academic burden, respectively. Thus, a targeted investigation of the academic burden of higher education students is required, along with the development of effective investigation tools, so that the levels of the burdens in basic and higher education can be compared and discussed under a unified framework that accounts for students’ specific physical and mental development.

### 2.3. Progress and Limitations of Research into Subjective Academic Burden

Research focusing on negative achievement emotions encompasses investigations of subjective academic burden. The concept of achievement emotion was first explicitly proposed by Pekrun et al., and is defined as the linking of various emotions, such as joy, hope, pride, relief, anger, anxiety, shame and guilt, hopelessness, and boredom, to learning and achievement [36]. This concept has since attracted extensive academic attention [37,38], and a series of questionnaires aimed at effectively investigating such emotions in various groups and situations have been developed. The initial Achievement Emotions Questionnaire (AEQ) [39,40] has been adapted for middle school [41,42] and elementary school (AEQ-ES) [43,44], and the Test Emotions Questionnaire (TEQ) [45,46,47] and the Achievement Emotions Questionnaire-Mathematics (AEQ-M) [48] have also been developed. Most questions about subjective academic burden in these questionnaires are situated after the achievement emotion questions, and most surveys focus on students’ negative psychological experience rather than both their negative and positive emotions.

We regard this approach as a limitation of the questionnaires for the following reasons. First, current research has demonstrated that positive and negative emotions are two relatively independent dimensions, rather than two poles of the same dimension. The premises and results of these two emotional forms are different, so they must be measured separately [49,50,51]. Second, an individual’s emotional state can be distinguished into immediate and holistic emotional experiences. Holistic emotional experiences are only gained over a period of time (ranging for example from a day to a year), which involves a collection of several immediate positive and negative emotions [52]. At any point in time, the negative emotions will be negatively correlated with positive emotions, but the overall correlation between positive and negative emotions will be greatly reduced over a sustained period of time [53]. Measuring subjective academic burden is clearly an evaluation of overall emotional experience. Some bias may be involved in using questionnaires to ask participants to report their overall emotional experiences over time based on their specific experiences at a certain point. This involves introspective and retrospective evaluation, but the integrated evaluation of emotional experience at different moments is not equal to the simple average of all such moments. For example, some construction may be involved in the recall process of individuals using a self-report scale to evaluate their subjective academic burden. Unpleasant experiences may later be interpreted and processed positively [54], and individuals’ answers may be easily affected by the situation or their emotional states at the time [55]. The “peak-end” rule also suggests that when individuals judge their overall emotional experiences over time, they only pay attention to the peak and the end of the experience and ignore the process [56]. Socially acceptable responses can also cause errors in the self-report scale [57]. Thus, other measurement methods should be actively explored in the study of subjective academic burden to address the possible shortcomings of the single self-reported questionnaire.

The measurement methods of subjective wellbeing (SWB) can be applied to studies of subjective academic burden, as both are types of holistic emotional experience. Effective SWB measurement methods include questionnaires designed for various groups of Chinese students [58,59,60] and non-questionnaire methods, such as peer evaluation, recording non-verbal behavior, physiological measures (e.g., cortisol), cognitive measures (e.g., the implicit association test), the day reconstruction method (DRM), the experience sampling method (ESM), ecological momentary assessment (EMA), and the U index [61,62]. Many of these measures have been proven to have high reliability and can be applied as useful supplements in assessments of subjective academic burden.

The Word Association Test (WAT) that we apply is a classic implicit cognitive measurement method first devised by Francis Galton in 1879 and later applied by Wilhelm Wundt, Emil Kraepelin, Carl Gustav Jung, and other renowned psychologists [63]. SWB has been found to have a certain stability [50], and the varying levels of SWB may be due to long-established differences between individuals. The Implicit Association Test (IAT) has been used to measure SWB [64]. Thus, we assume that subjective academic burden also has a certain stability and that various long-term differences in the learning concepts of students with high and low academic burdens are likely. The WAT can be used to investigate the subjective and objective academic burdens of basic and higher education students, which lays the foundation for a comparison and discussion of students’ academic burden under a unified framework based on their physical and mental development. In addition, as an implicit measurement method, the WAT can effectively avoid any bias caused by socially desirable responses. In this study, we use data previously collected through a large-scale WAT-based survey of the learning concepts of students from primary school to university level in Guangdong Province in 2019 [9,10]. This enables a bottom-up analysis, and we aim to provide an overview of Chinese students’ academic burden and explore its influencing mechanisms, thus informing further research in this field and the formulation of relevant policies.

## 3. Method

### 3.1. Participants

We recruited 2326 Chinese students from ordinary primary schools, junior and senior high schools, and universities in medium-sized cities in Guangdong Province, covering Grades 1 to 15. Each three adjacent grades were divided into the five learning groups of primary school lower grade, primary school higher grade, junior high school, senior high school, and a university group. The numbers of participants in each group were 460, 520, 472, 411, and 463, respectively, and were balanced in terms of gender [9,10].

### 3.2. Process

We first applied the WAT, and the participants took “learning” as the target word for free association and wrote 20 terms related in any way to it, including words, phrases, idioms, sayings, epigrams, or poems. We did not require the primary school groups to provide a minimum number of terms, but we imposed a time limit of 10 min. Data for the university group were collected using a questionnaire derived from Wenjuanxing, a Chinese online survey platform, and those in other grades completed paper questionnaires. Nvivo 11.0 was then used for cluster coding associative terms. After deleting invalid words that were not closely related to learning, three coders classified associative terms with the same or similar semantics, and 31 category indicators were obtained. The frequency of each category indicator was calculated according to gender and grade (or learning group), and the frequency was divided by the total number of associative terms per gender in the grade. The higher the frequency, the more learners attached importance to this category indicator (see [9,10] for the detailed process).

### 3.3. Data

We mainly focused on three categories related to academic burden: “objective academic burden”, “negative achievement emotion”, and “positive achievement emotion”. Objective academic burden included typical associative terms, such as “*exam (考试),*” “*test paper (试卷),*” “*assignment (作业),*” “*grade (成绩),*” “*GPA (绩点),*” “*academic credit (学分)*”, “*ranking (排名),*” “*after-school tutor (补习)*”, “*flunk (挂科),*” “*exam-oriented education (应试教育)*”, and “*criticism (批评)*”, etc.; the negative achievement emotions included “*boring* *(无聊),*” “*arduous* *(辛苦),*” “*tired (累),*” “*difficult (难),*” “*tired (烦),*” “*decadent* *(颓废)*”, “*struggle (挣扎),*” “*entangled (纠结)*”, “*hate (讨厌)*”, “*stress (压力)*”, and “*dull (枯燥)*”, etc. The positive achievement emotions included “*like (喜欢),*” “*happy (快乐),*” “*joyful (开心),*” “*enjoyable (享受),*” “*confident (自信)*”, “*delight (乐趣),*” “*interesting (好玩),*” “*sense of achievement (成就感),*” “*excited (兴奋)*”, and “*find pleasure in it (乐在其中)*”, “*no sweet without sweat* *(先苦后甜)*”, etc. The connotations of the three indicators are consistent with those in the reviewed research, as these associative terms suggest.

The terms most frequently mentioned in our data [9,10] were those in the “learning attitude” category, which included the eight secondary indicators of “active and independent”, “diligent and hard-working”, “industrious and persistent”, “modest and studious”, “earnest and attentive”, “time-cherished”, “rigorous and meticulous”, and “lifelong learning”. We thus consider these to be factors that influence academic burden.

Table 1 shows the total number of associative terms collected in the five learning sections and the frequency of each category.

The terms most frequently mentioned in our data [9,10] were those in the “learning attitude” category, which included the eight secondary indicators of “active and independent”, “diligent and hard-working”, “industrious and persistent”, “modest and studious”, “earnest and attentive”, “time-cherished”, “rigorous and meticulous”, and “lifelong learning”. We thus consider these to be factors that influence academic burden.

Table 1 shows the total number of associative terms collected in the five learning sections and the frequency of each category.

Thus, WAT is an implicit cognitive measurement method based on overall emotional experience, and it reveals that students’ positive and negative achievement emotions are significantly and moderately positively correlated (*r* = 0.56, *p* = 0.01), which is consistent with our hypothesis in the above review. Over time, students’ negative and positive achievement emotions may therefore coexist. Students have a complex emotional experience involving “love and hate” and “mixed feelings” about learning. Students’ engagement in learning to some extent increases with the intensity of the emotional experience. Thus, we assume that only when the total level of negative achievement emotion is greater than the positive level over time can students be regarded as perceiving a subjective academic burden. Thus, we take the difference between the frequencies of negative achievement emotion and of positive achievement emotion as the index of subjective academic burden.

## 4. Results

### 4.1. An Overall View of Students’ Academic Burden

We first compare and discuss the academic burden of basic and higher education students under a unified framework to provide an overall view of students’ academic burden in China.

Table 1 above reveals the frequencies of the category indicators and their changing trends throughout the stages of education. The undesirable dimensions of objective academic burden and negative achievement emotion are generally more prominent than the desirable dimension of positive achievement emotion, which indicates that students have a heavy academic burden. Both objective and subjective academic burden increase with the educational stages and reach a peak in the university stage, with the fastest development observed in junior high school. However, the learning attitude shows a opposite trend, falling from the higher grade of primary school and reaching the lowest point in the university stage. The results of the independent sample F-tests are statistically significant, indicating that the differences between school stages are unlikely to be caused by sampling fluctuations. In addition, the results of the T-test show no significant gender differences in the four categories, and thus the effect of gender is not considered in the following analyses.

**Figure 1:**
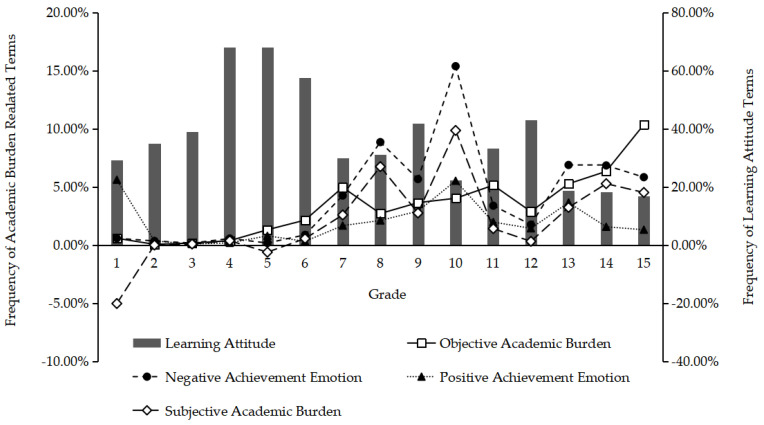
The trend of the frequency of each category by grade.

Figure 1 shows the trend by grade, which illustrates in a more detailed and intuitive way the trends of these four categories and their relationships.

Figure 1 clearly shows that as the grades progress, most of the inflection points of the indicators fall on the transitional grades in the school stages, which indicates that the psychological adjustment required when students change their learning environment may be an important factor affecting the academic burden. We also observe three other interesting phenomena.

First, we can assume that students face more enrollment pressure in, for example, Grades 6, 9, and 12. However, the objective and subjective academic burdens appear to decline while the learning attitude increases compared with adjacent grades, which indicates that to some extent, students may be able to cope well with the enrollment pressure and actively self-adjust.

Second, students appear to be full of interest, enjoyment, and enthusiasm for learning when they first attend a new school because the three positive achievement emotion peaks are in Grade 1, Grade 10, and the freshman year. However, their passion diminishes with the increase in study time. This may reflect the problem in the education system that the teaching content or method cannot effectively stimulate students’ learning, and thus their learning will not develop sustainably.

Third, the data show that university students face greater objective and subjective academic burdens compared to those in basic education, which is not consistent with our intuition. However, we also observe that university students have the worst learning attitudes and that their negative achievement emotion ranks second. This indicates that university students suffer from serious learning fatigue, and so effective intervention is urgently required.

### 4.2. The Influencing Mechanism of Students’ Academic Burden

Our review suggests that researchers have paid particular attention to the influence of objective signs of academic burden on subjective academic burden (i.e., negative achievement emotion). However, the results of these previous studies are inconsistent. Some have found a significant positive correlation between the two factors [65], while others have failed to find a significant correlation [66]. This indicates that the route by which objective academic burden influences students’ subjective academic burden may be affected by other factors, and thus the conditions under which the objective academic burden will lead to negative achievement emotion remain unclear [67]. We further explore this influencing mechanism by using objective academic burden as an independent variable and subjective academic burden as obtained through the difference between negative and positive achievement emotion as a dependent variable. Thus, we examine whether learning attitude potentially regulates or mediates the relationship. Table 2 below shows the correlation between objective and subjective academic burdens and learning attitude.

**Figure 2:**
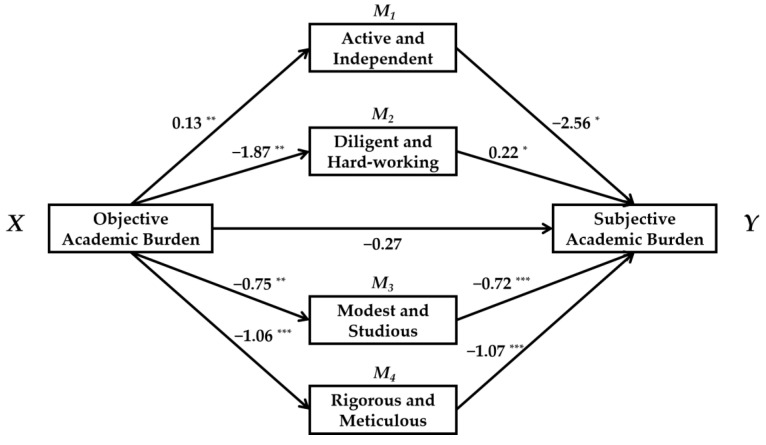
Mediating effects of secondary indicators of learning attitude. * *p* < 0.05, ** *p* < 0.01, *** *p* < 0.001.

First, we tested the direct effect, and we found that the resulting regression coefficient of subjective academic burden on objective academic burden was significant (*c* = 0.63, *t* = 2.90, *p* = 0.007). Learning attitude was then used as a moderating variable to test its moderating effect. The effects of objective academic burden on subjective academic burden were the same at different levels of learning attitude, and thus the moderating effect was not significant. Next, learning attitude was tested as a mediator variable, and Model 4 (a simple mediation model) was applied in SPSS macro. We found that the mediating effect was not significant: objective academic burden had a significant negative predictive effect on learning attitude (*a* = −3.81, *t* = −4.12, *p* = 0.003, 95%BootCI = [−5.70, −1.91]); however, learning attitude had no significant predictive effect on subjective academic burden (*b* = −0.05, *t* = −1.21, *p* = 0.24, 95%BootCI = [−0.14, −0.04]), and the mediating effect (*ab* = 0.20) included 0 in the 95% confidence interval ([−0.081, 0.560]) calculated through bootstrap sampling. The category of learning attitude contained eight secondary indicators, and thus their counteracting effects may have led to the outcome that the mediating effect of the mean value of learning attitude was not significant. Thus, to further refine the results, we added these eight secondary indicators into the model together to test the parallel multiple mediating effects. The results are shown in Table 3 and Figure 2. The mediating effects of the four indicators of active and independent, diligent and hard working, modest and studious, and rigorous and meticulous were significant (the 95% confidence interval does not include 0). As the direct effect was not significant, the other four indicators of learning attitude thus played fully mediating roles. The results therefore suggest that students will take positive measures when their objective academic burden increases. However, they will be less serious, studious, and diligent. Positive coping can relieve the feeling of being subjected to academic burden, but the decrease in engagement will lead to further negative psychological experiences.

## 5. Discussion and Supplementary Investigation

### 5.1. Trend of Academic Burden by Grade

The results of our large-scale empirical investigation based on the WAT reveal that objective and subjective academic burdens (measured by either negative achievement emotion or the difference between negative and positive achievement emotion) of primary and high school students in Guangdong Province increases with the stage of education. This finding appears reasonable, as higher-graders encounter more subjects, more complicated curriculum content, more intense competition with peers, and more expectations and emphasis on the college entrance examination within the Chinese context. This finding has also been repeatedly and consistently confirmed from various perspectives in other studies (see [16,18,21,23,65,68,69,70,71,72,73,74,75,76,77,78,79]), which indicates that excessive academic burden is a serious problem for primary and high school students in China. The consistent results also validate the WAT as an effective method of measuring academic burden.

A major innovation of this study is that it is the first to longitudinally compare the academic burden of basic and higher education students under a unified framework, which has important implications in the context of the current national education policy environment. The results indicate that university students bear a greater academic burden than primary and high school students in China, particularly in terms of the objective viewpoint, which is almost twice that of primary and high school students. Why does this go against popular intuition? To avoid any sampling bias, we conducted a random survey of about 400 people with a simple questionnaire to confirm the findings of the WAT, as shown in Table 4. We found that nearly three-quarters of the university students perceived their academic burden at university to be heavier than that in senior high school, and this proportion did not differ significantly in terms of gender, types of schools, majors, or academic achievement. However, graduating students facing employment or further study are significantly more likely to report that their senior high school academic burden was heavier, which suggests that the perceived and reported academic burden is indeed due to school curriculum tasks. These results again confirm our conclusions derived from the WAT.

We conducted further small-scale interviews to provide a deeper understanding of the situation. The interviewees said they had more “freedom” after attending university, but “freedom” does not mean more “relaxation”. Professional courses, such as in medicine, engineering, science, and other majors, are more difficult, and much of the learning content is complex. However, students taking these courses are more likely to encounter more outstanding peers from all over the country. The pressure of peer competition is even greater if high-level academic achievement is an expectation, such as scholarships or post-graduate qualifications. Some interviewees joked that “I want to return to my senior year in high school and take a break”, or “It’s the biggest lie to say ‘you’ll be relieved when you go to university.’” Such responses suggest that the increased academic burden at university is a continuation of basic education, as the main underlying reasons are virtually the same: increased learning difficulty, heightened peer competition, and consistently high academic goals.

However, the data derived from the WAT suggest that the learning attitudes of university students are at extremely low levels, below those of half of the primary and high school students, which reflects a situation in which university students do not sufficiently engage in their studies. The interviews also suggest that university students are polarized in their attitudes toward academic pursuit. When entering university, some held the mistaken perception that “going to university is just for ‘fun”, and they have the “firm determination” that they “will ‘kill time’ for four years”. Others said they are really not interested in their major and have tried hard but failed, and thus they ended up “lying flat”. These results indicate that a more precise management classification of academic burden is required.

Very few empirical investigations have been conducted into the academic burden of university students in China. In addition, many of the related studies on student engagement focused only on its universality and lack detailed discussions of the differences among and between groups. Thus, any conclusions about whether university students should “increase their burden” are premature, and further empirical investigation is required.

### 5.2. The Effect of Learning Attitude on Objective and Subjective Academic Burden

Learner-oriented and centered personalized education has become a focus of education [80]. Current educational reform typically considers the individual differences of students, and it has clarified the non-linear relationship between student engagement (objective academic burden) and subjective academic burden and has advocated “scientific burden reduction” over a “one-size-fits-all” approach. Zhang et al. [17] divided students into four types: “low engagement and high burden”, “low engagement and low burden”, “high engagement and high burden”, and “high engagement and low burden”. They noted that “students’ burden cannot be generalized, as some need to reduce burden, some need to increase time engagement, and some need to improve their learning quality” and that “The core of burden reduction lies in improving students’ learning quality and other non-academic qualities. Students with good learning quality usually have a lower burden feeling regardless of their engagement in learning”. Here, learning quality includes the important factor of attitude toward learning. Fang et al. [67] found that learning attitude can play an important role in regulating the relationship between subjective and subjective workload. They noted that “compared with students with good learning attitude who can cope with the negative effects of objective academic burden relatively effectively, students with poor learning attitude are more likely to be affected by objective academic burden, thus bearing a heavier subjective burden” and that “the change of students’ learning attitude is one of effective ways to reduce academic burden”. We also explored the influence of learning attitude, and although our results are similar, we identified the mediating effects of learning attitudes on the relationship between objective and subjective academic burden through the WAT. However, our findings do suggest that learning attitude plays an important role in students’ academic performance. Thus, in terms of either “reducing the burden” in primary and secondary school or “increasing the burden” in university, we should not focus exclusively on the “quantity” of students’ academic burden, but should also consider improving students’ “learning quality”, particularly through research and interventions focused on non-intellectual factors.

## 6. Conclusions, Limitations, and Implications

### 6.1. Conclusions

The main points, core research findings, and innovations of this study are summarized as follows.

(1) The measurement of subjective academic burden should simultaneously focus on both negative and positive emotional experiences over time, and various methods developed in SWB research can be used for reference.
(2) The results demonstrate that the academic burden of primary and high school students in China increases with the academic stage, which confirms the necessity of reducing the academic burden in basic education.
(3) This study is the first to compare the academic burdens of basic and higher education students under a unified framework. The results demonstrate that the university students face more of a burden than primary and high school students, but their learning attitudes are less positive. This finding can inform and inspire policy makers considering reasonable “burden increases” for university students.
(4) Students’ academic burden, emotions related to achievement, and learning attitudes were found to fluctuate greatly during the transition periods of school stages, which suggests that we should provide guidance for students that enables them to psychologically adjust in these periods.
(5) Learning attitude was found to play a mediating role in the relationship between objective and subjective academic burden. Students can actively cope with an increase in the objective academic burden, but their engagement will be less serious, studious, and diligent, which can lead to an increase in their subjective academic burden. This suggests that considerations of “reducing the burden” or “increasing the burden” should focus more on improving students’ learning quality.

### 6.2. Limitations and Future Research

This study has some limitations. First, our research is a bottom-up analysis of data obtained through our previous work, so we only explore how learning attitudes affect academic burden. Other important influential factors should be addressed in the future. Second, the WAT data sample was confined to Guangdong Province, so the results may not be generalizable to the whole country, and thus investigations in other areas and different groups should be conducted. Finally, this is the first time the WAT has been used to measure students’ academic burden, so further study from other research teams jointly evaluating the effectiveness of this method could be beneficial.

Moreover, our study proposes a new research direction in the field of academic burden. Studies in China have begun to consider academic burden in the last century, but more recent research in this field has not made significant progress. The two main problems are that first, no consistent understanding or definition of academic burden and its extension have been achieved, and thus no definitive research theory has been developed, and second, academic burden is still mainly measured through questionnaires, and no other effective measurement methods have been applied. We explore other methods that can inform the questionnaire approach. The American physicist Bridgman proposed that if scientific terms or concepts are to avoid ambiguity, they should be defined by the operational methods used in measurements [81]. Consensus can be reached and the comparability of the research results can be improved by exploring diverse measurement methods of academic burden and discussing their operational definitions in empirical studies. In the review section of this study, we propose that the study of subjective academic burden can apply the measurement methods of SWB, including peer evaluation, non-verbal behavior recording, physiological measures, cognitive measures, DRM, ESM, EMA, and the U index. In addition, our study is the first to use the WAT as a cognitive measurement to investigate the academic burden of students from primary schools to universities. However, the validity of the results obtained through this novel approach should be further verified. We also note that the WAT is not an independent and specific measurement method for academic burden, as the data collection process is not efficient and the sorting and analysis are complex. This study provides possibilities and inspiration for further studies of academic burden through a bottom-up analysis of our previous data. The measurement of academic burden can now be further combined with big data and artificial intelligence technology. Establishing a scientific and accurate diagnosis system for students’ academic burden, applying personalized burden reduction programs for different students, and using intelligent and adaptive learning systems to improve their learning quality will be of benefit [17].

### 6.3. Implications for Education Policies

Based on our findings, we offer some specific suggestions for the formulation and implementation of China’s education policy. First, we should improve interventions concerning students’ learning attitudes and consider how to improve the quality of their learning. The current policy of increasing or reducing academic burden has not yet fully considered “improving students’ learning quality”, which should be its primary objective. A singular focus on burden intervention will not typically achieve desirable outcomes. Consistent with previous studies [17,67], our study reveals that non-intellectual factors, such as learning attitudes, willpower, and learning methods and strategies play an important role in reducing students’ subjective academic burden and that the phenomenon of students’ “relaxation in universities” may be a result of an insufficient engagement with learning attitudes. China has adhered to “virtue-oriented” learning beliefs since ancient times, and it attaches great importance to the cultivation of learning attitudes. Good attitudes play a central role in stimulating the initiative of Chinese learners and enables them to excel in international standardized tests to some extent. This represents a unique advantage for learners in East Asian Confucian cultures [82]. Our study reveals that the learning attitudes of university students in China have experienced a major decline, which merits the attention of relevant government departments. The underlying reasons should be closely examined and active interventions should be implemented. Intervening to support university students in developing positive learning attitudes can be one method of rationally “increasing the burden”.

Second, we should improve the mental health education students that receive and focus on the psychological adjustment of those in entrance and graduation grades. Our results suggest that psychological maladjustment in the transitional grades between school stages may be an important factor that increases academic burden, and it can be used to predict the burden of university students. After entering university, high school graduates are affected by sudden changes in life and study and are likely to have new and negative psychological experiences that will lead to self-doubt and negation of their previous concepts, and thus to the weakening of their “virtue-oriented” learning beliefs (the most important manifestation being the decrease of engagement) [9,10]. Thus, active and effective mental health education is necessary to provide guidance on psychological adjustment and pressure management for freshmen and graduates in each education stage and to help them get through the “transition period with sudden change” as quickly as possible.

Third, scientific monitoring and the classification-based management of academic burden should be implemented to avoid a “one size fits all” approach. Individual students differ, and education in this new era should be tailored to their various levels of aptitude. Various studies have called for the implementation of classified management and precise policies concerning the academic burden of primary and secondary school students to improve the effectiveness of burden reduction policies [17,83,84,85,86]. This study pays special attention to university students and challenges the perception that their academic burden should be increased. Critical conclusions about the policy should be drawn with caution, considering the restricted survey sample and the newly developed survey instrument, but a rigid policy of increasing the burden should not be encouraged. We expect the relevant departments to introduce more rigorous, precise, and scientific education policies after thorough investigations. As mentioned, the development of new techniques that apply big data and AI can achieve accurate and real-time diagnoses of academic burden and thus provide personalized guidance to improve students’ learning quality.

## Figures and Tables

**Table 1:** Total number of associative terms collected in the five learning sections and the frequency of each category.

	Primary School Lower Grade	Primary School Higher Grade	Junior High School	Senior High School	University	*M*	*SD*
Total number of associative terms	2045	4068	9589	8106	8394	6440.40	3219.17
Frequency of objective academic burden	0.18%	1.69%	3.85%	3.94%	7.13%	3.26%	2.71%
Frequency of negative achievement emotion	0.32%	0.64%	6.13%	7.46%	6.59%	4.07%	4.23%
Frequency of positive achievement emotion	1.06%	0.43%	2.19%	3.16%	1.95%	1.97%	1.78%
Frequency of subjective academic burden	−0.73%	0.21%	3.94%	4.30%	4.64%	2.47%	2.53%
Frequency of learning attitude	35.44%	61.79%	34.00%	32.37%	18.08%	36.45%	16.83%

**Table 2:** The correlation coefficient between the four category indicators.

	Objective Academic Burden	Subjective Academic Burden	Learning Attitude
Objective academic burden	-		
Subjective academic burden	0.48 **	-	
Learning attitude	−0.61 **	−0.45 *	-

* p < 0.05, ** *p* < 0.01.

**Table 3:** Mediating effects of secondary indicators of learning attitude.

	Mediator Variable	Effect	BootSE	BootLLCI	BootULCI
Direct effect	/	−0.27	0.21	−0.71	0.17
Indirect effect	Active and independent	−0.32	0.21	−0.83	−0.05
	Diligent and hard-working	−0.40	0.36	−1.34	−0.07
	Industrious and persistent	0.06	0.15	−0.09	0.42
	Modest and studious	0.54	0.24	0.12	1.03
	Earnest and attentive	1.13	0.63	0.45	2.79
	Time-cherished	0.16	0.15	−0.07	0.50
	Rigorous and meticulous	−0.11	0.10	−0.31	0.07
	Lifelong learning	−0.16	0.15	−0.54	0.01

**Table 4:** Results of a simple questionnaire survey on university students’ academic burden.

Basic Information			Heavier Academic Burden in University	Lighter Academic Burden in University	Differences Test
Items	Options	Percent of Cases	Percent of Cases	Percent of Cases	Chi-Square Value (*p* Value)
Gender	Male	42.79%	67.4%	32.6%	3.680 (0.055)
	Female	57.21%	76.1%	23.9%	
Types of school	Key universities (985/211)	20.15%	72.8%	27.2%	1.247 (0.742)
	The first batch of undergraduate	43.28%	74.7%	25.3%	
	The second and third batch of undergraduate	30.6%	69.9%	30.1%	
	Technical/vocational college	5.97%	66.6%	33.4%	
Types of major	Science and engineering	51.49%	72.5%	27.5%	0.001 (0.972)
	Liberal arts	48.51%	72.3%	27.7%	
Grade	Freshman	6.72%	66.7%	33.3%	15.858 ** (0.003)
	Sophomore	40.8%	74.3%	25.7%	
	Junior	47.51%	74.8%	25.2%	
	Senior	3.98%	50.0%	50.0%	
	5th year in university	1%	0%	100%	
Specialist ranking	Top 25%	39.8%	74.4%	25.6%	4.521 (0.104)
	Middle 25–75%	53.98%	55.4%	44.6%	
	Bottom 25%	6.22%	88.0%	12.0%	
Failure in course	Yes	19.4%	79.5%	20.5%	2.440 (0.118)
	No	80.6%	70.7%	29.3%	
Total		402 (100%)	291 (72.4%)	111 (27.6%)	—

** *p* < 0.01.

## Data Availability

The data presented in this study are available upon request from the corresponding author because of privacy and ethical restrictions.
